# Transitions: Experiences of younger persons recently diagnosed with Alzheimer-type dementia

**DOI:** 10.1177/14713012231155516

**Published:** 2023-02-02

**Authors:** Malin Aspö, Leonie Visser, Miia Kivipelto, Anne-Marie Boström, Berit S Cronfalk

**Affiliations:** Department of Neurobiology, Care Sciences and Society, Division of Clinical Geriatrics, 27106 Karolinska Institutet, Sweden; Theme Inflammation and Aging, Karolinska University Hospital, Sweden; Department of Neurobiology, Care Sciences and Society, Division of Clinical Geriatrics, 27106 Karolinska Institutet, Sweden; Department of Medical Psychology, 522567 Amsterdam University Medical Center, University of Amsterdam, The Netherlands; Department of Neurobiology, Care Sciences and Society, Division of Clinical Geriatrics, 27106 Karolinska Institutet, Sweden; Theme Inflammation and Aging, Karolinska University Hospital, Sweden; Department of Research and Development, Stockholms Sjukhem Foundation, Sweden; Theme Inflammation and Aging, Karolinska University Hospital, Sweden; Department of Research and Development, Stockholms Sjukhem Foundation, Sweden; Department of Neurobiology, Care Sciences and Society, Division of Nursing, 27106 Karolinska Institutet, Sweden; Department of Neurobiology, Care Sciences and Society, Division of Nursing, 27106 Karolinska Institutet, Sweden; Department of Health Sciences, 7666 Red Cross University College, Sweden

**Keywords:** early-onset dementia, young-onset dementia, Alzheimer’s disease, dementia, qualitative research, transitions theory, Afaf Meleis

## Abstract

Receiving a diagnosis of dementia before the age of 65 has a huge impact on everyday life. Previously, the disease trajectory has mainly been described from the perspective of older persons. However, young persons with dementia are confronted with specific challenges, influencing the type of life-changing events, or ‘critical points’ that they may experience. The aim of this study was therefore to describe experiences of persons recently being diagnosed with young-onset dementia. In total, 14 participants with dementia due to Alzheimer’s disease (10 woman/4 men) with an average age of 59 were included in the study. Interviews were conducted within 2 months after receiving the diagnosis and analyzed using qualitative content analysis with an inductive approach, resulting in three categories: (1) *A life changing moment*, (2) *An ongoing process,* and (3) *Remaining in control.* The findings show that receiving such a diagnosis was experienced by participants as a life changing moment, followed by them seeking to come to terms with the diagnosis and reflecting on its meaning, in which various strategies were adopted to remain in control. The current study highlights three critical points considering the diagnosis of young-onset dementia that warrant special attention and provides insight into factors related to delay in healthy transitioning after receiving the diagnosis, as well as factors that may facilitate successful transitions.

## Background

The estimated prevalence of dementia worldwide is over 50 million and expected to triple by 2050 (Alzheimer´s disease International, 2019). In Sweden, between 130.000 and 150.000 persons are living with dementia (Region Stockholm, 2019) and approximately 9.000 were diagnosed before the age of 65 (Socialstyrelsen, 2014). Persons who develop dementia before the age of 65 are commonly referred to as persons with young-onset dementia. A recent review by Hendriks et al., (2021) stated the global prevalence of dementia among persons aged 30–64 to be 119 per 100.000. The prevalence of young-onset dementia appears to be slightly higher among woman, even if information regarding gender differences still is sparse (Kvello-Alme et al., 2019).

Previously, the disease trajectory has mainly been described from the perspective of older persons, identifying various critical points such as recognizing early symptoms, initiating contact with health care services, the diagnostic process, disclosure of diagnosis and support after diagnosis (Samsi & Manthorpe, 2014). Further, loss of driver’s license, financial issues, changing care settings, and the occurrence of behavioral symptoms are described by Johannessen et al. (2019) and Rose and Palan Lopez (2012) as life changing events. In addition, decisions regarding if and when a person could tell others about their disease should also be considered as critical points (Milby et al., 2015).

Young persons with dementia are confronted with specific challenges, influencing the type of critical points that they may experience. As such, receiving an accurate diagnosis has been described as a challenge by Sansoni et al., (2016). They put forward that the time between noticing the first symptoms and receiving a dementia diagnosis appears to be twice as long for a younger person, compared to an older person. This is mainly due to the misconception that the person is too young to have developed dementia, or that their symptoms are assumed to be caused by stress or burnout (Sansoni et al., 2016). In addition, most persons younger than 65 are unprepared for the diagnosis, since they also have difficulties identifying themselves with the stereotype of being a person with dementia, that is, an old, frail, and dependent person (Clemerson et al., 2014; Sawchuk, 2015). Millenaar, de Vugt, et al. (2016) suggest that being diagnosed with young-onset dementia could significantly impact persons’ everyday life as they might still participate in the workforce and, some of them, also provide for a family (with children). Following the diagnosis, the person needs to adjust to the new situation, which includes managing changes in identity and roles professionally and privately, and dealing with losses, such as loss of employment and independence (Harris, 2004; Sansoni et al., 2016). The premature ending of working life after receiving the diagnosis has been described as an important critical point and it is essential to address the unwanted ending of working life as a life-changing event.

Consequently, persons with young-onset dementia need (new) coping strategies for managing daily life. For example, reevaluating priorities in life and focusing on activities that are experienced as positive and meaningful to improve a sense of wellbeing (Robinson et al., 2011). Cabote et al. (2015) and Sansoni et al. (2016) argue that with the right support during the recurrent phases of change foreseeable to a neurodegenerative condition such as Alzheimer’s disease, the person may sustain independence and remain in control of their own lives for longer. However, persons with young-onset dementia often experience difficulties getting referred to available services, which hinders them from receiving and accepting support (Sansoni et al., 2016). Beattie et al. (2004) put forward that the lack of age-appropriate services could result in persons rejecting the services and support on offer, as their self-image is that they are too young.

Meleis’ Transitions Theory is valuable framework to better understand a person’s needs in relation to health and illness (Meleis et al., 2000; Rose & Palan Lopez, 2012). According to Meleis et al. (2000) a transition is initiated when a person shows awareness about a life-changing event, that is, a critical point. A transition is associated with change, takes place over time, and may comprise a shift in how a person perceives their identity, various roles, health status, abilities, and/or relationships. A first indication of change may include signs of anticipation and changed awareness, that is, a person’s perception, knowledge, and recognition of the transition. Such a shift in awareness could result in periods of instability or distress, in which the person is at risk of making unhealthy decisions. Alternatively, awareness could generate active engagement in the process of transitioning, for example, by searching for information, preparing for the process, and making preventive changes (Meleis et al., 2000).

By recognizing critical points and changes, and subsequently stimulating or supporting active engagement in the transition process, health or social care staff can play a pivotal role in facilitating successful and healthy transitions. The aim of this study was therefore to describe experiences of persons recently being diagnosed with young-onset dementia.

## Methodology

### Study Design

This interview study is part of a longitudinal study with a qualitative approach, focusing on experiences of persons with young-onset dementia.

#### Setting

Persons were recruited from two specialized memory clinics in Sweden. Persons with a suspected cognitive disease, many relatively young, are referred to these clinics after undergoing a basic cognitive evaluation by for example their general practitioner. Approximately 600 patients are seen by these clinics yearly. Out of these, about 50 persons younger than 65 are diagnosed with dementia.

#### Participants

The study employed a consecutive sampling strategy based on the following inclusion criterion; persons ≤65 years of age recently diagnosed (≤2 months) with dementia but no previously diagnosed condition of impaired cognition, Mini mental state examination (MMSE) score ≥24, able to communicate verbally and in writing, and to provide verbal and written consent.

#### Procedures

After receiving the diagnosis, a physician at the clinic informed all eligible persons about the study. If a person expressed interest to participate, he/she was informed that the first author would contact them. The first author contacted the persons by a phone call, to introduce herself and explained the purpose of the study. This was followed by a verbal consent and a time for the interview was arranged.

#### Data collection

Participants were recruited and interviewed between September 2018 and February 2020. The interviews were conducted within 2 months after diagnosis and took place at a location chosen by the participant, most frequently at the memory clinic. The interviewer highlighted that the interview was conducted as part of research and that information provided by them would not be shared with the clinical team. Participants were asked to describe onset of symptoms, the diagnostic process, reactions when receiving the diagnosis, experiences of the impact of diagnosis, and any critical points. Table 1 displays example questions from the interview guide. The interviews lasted between 45 and 90 minutes and were audio recorded and transcribed by the first author who is a registered nurse, specialized in care of elderly, with extensive experience of persons with dementia. To pilot the interview guide, the first two interviews were conducted together with the last author. These interviews were also included in the analysis. In addition to the interview participants were also asked to complete a survey collecting information regarding gender, education, employment status, marital status and if they had any children.

**Table 1.** Example questions from the interview guide.

**Onset of symptoms**
Can you please describe how it all started?
• When did you first notice symptoms that got you worried?
• What were the symptoms and how did you interpret them?
• When/why did you get in contact with a physician regarding your symptoms?
**Diagnostic process**
Can you please tell me about the procedure of being transferred to the memory clinic?
• What were your expectations of the evaluation?
• Did you know why you had been referred to the clinic?
• How was your experience of the procedures?
**Diagnosis**
Tell me about the day you came back to the clinic to get the results from your memory evaluation?
• What were your thoughts when coming to the clinic?
• How did you react when being told about the diagnosis?
• Was the diagnosis expected?
**Impact of diagnosis**
It has now been X weeks/months since you were told about your diagnosis. How has this affected your daily life (work, family, socially)?
• How is your daily life today, compared to before?
• How does your symptoms affect your daily life?
• How has the diagnosis impacted you on a personal level?
• What are your thoughts about the future?
**Critical points**
Looking back, are there any distinctive points or periods that you have experienced as critical since you first noticed symptoms?
• What have been hardest/most difficult?
• What have been positive?

#### Analysis

Qualitative content analysis with an inductive approach was performed (Elo & Kyngäs, 2008; Graneheim & Lundman, 2004). The procedure of analysis started by reading all interview transcriptions several times to get an overall understanding. This was followed by identifying units of meaning describing experiences related to living with dementia*.* These units were condensed, coded, and arranged into unique sub-categories, which in the final step were grouped into categories. Table 2 illustrates the analysis process. The analysis was conducted separately by the first author and the last author, followed by discussions concerning similarities and differences to reach consensus regarding sub-categories and categories. The authors have extensive knowledge and experience of persons with dementia and dementia care and to lessen the influence of the authors’ pre-understanding, especially regarding critical points, all authors have actively reflected upon their own pre-understanding (Graneheim et al., 2017).

**Table 2.** Example of the analysis process.

Units of meaning	Codes	Sub-categories	Categories
Yes, that would be hard. It wouldn’t work either. If so, they would understand it after a while. Then it becomes of those things you don’t talk about. And it’s kind of nice that it...is public to those closest to me, then they can tolerate more. How one behaves or talks…	Reasons to tell	To tell or not to tell	Remaining in control
At first I did not really care if people knew or not. But then maybe you start to imagine that people might change attitude or maybe they will take me less seriously. That is worries I have but it’s not something I’ve noticed yet, but it is these kind of argument that goes into how you think about keeping it a secret.	Reflections on if to tell		
Some people throw themselves over it and read everything there is and so on. I’m not quite like that. Actually. But I think I have to, I’ll have to take it as it comes. I don’t want to read everything there is on the internet about this, because it can be difficult to sort the information	Not wanting to know	Information needs and preferences	
I mostly search for information that explain the disease and in this case I have found a special book about someone who thinks he has a method that works and also gives examples. So that it is kind of uplifting.	Information about the diagnosis		
I always put things in the same places, the first thing I do I put the keys in the key cabinet, the car key there, then I know. And then I put things in, and in the morning before I go to work, in the evening before I go to work I put out what I’m going to bring	Daily strategies	Everyday coping strategies	
Not that I feel there is a significant difference. But I have also learned to compensate, by say things in other ways (when not finding the correct words), I constantly compensate.	Compensatory strategies		

### Ethical Considerations

The study was approved by the Ethics Review Board in Stockholm, Sweden (Dnr: 2017/2400-31/1) and conducted in compliance with *The declaration of Helsinki* (World Medical Association, 2013). Written consent was obtained from all participants before any interview was conducted. Verbal and written information was provided repeatedly, emphasizing that participating was voluntary, and that the person could withdraw without further explanation at any time.

## Findings

In total, 14 participants participated: 10 women and four men, all diagnosed with dementia due to Alzheimer’s disease, with an average age of 59 and MMSE score of 27. Table 3 displays sample characteristics. Interviews were conducted within 2 months of participants receiving the diagnosis of dementia due to Alzheimer’s disease, reflecting on the participants own thoughts and experiences related to their recent diagnose. At the time of diagnosis and the interview, participants were in different phases of the disease, most had mild symptoms, that is they were still functioning independently for example still working and driving a car. The interview findings were clustered in three categories and eight subcategories, as displayed in Table 4.

**Table 3.** Sample characteristics.

		Mean (range)
Age		59 (49–65)
MMSE		27 (24–30)
		N*
Gender	Female	10
	Male	4
Education	High school	5
	University	9
Employment status	Not working due to dementia	6
	Employed full time	2
	Employed part time	3
	Retired	3
Marital status	Married, living together	10
	In a relationship, not living together	1
	Widowed	1
	Single	2
Children	Having children younger than 18	4
	Having adult children (older than 18)	10

Note: * In total, 21 persons were found eligible and asked to participate in the study during the inclusion period. Seven persons expressed no interest of participating in the study and were therefor not included.

**Table 4.** Sub-categories and categories.

Categories	Sub-categories
A life changing moment	First symptoms
	Personal and social life
	Working life
An ongoing process	Acceptance takes time
	Thoughts on existence
Remaining in control	To tell or not to tell
	Information needs and preferences
	Everyday coping strategies

### A Life Changing Moment

The participants described that to receive the diagnosis was a moment that changed their lives, with a personal and social impact. Most described that being diagnosed with Alzheimer’s disease at an early age was a shock. Most participants also still were employed when they first noticed symptoms.

#### First Symptoms

Difficulties at work was described as a first sign and symptoms were often first noticed at the workplace, either by the participants themselves or by colleagues. The participants described how work became more time-consuming as they needed to pay extra attention to perform tasks correctly. Problems with short-term memory and not being able to focus increased the fear of making mistakes.

*“It became difficult to perform my duties at work as my memory loss was getting worse. As I was in front of my computer… I could not remember what I saw and was supposed to do” (Participant 10, female).*

Some participants expressed that they feared early on that their symptoms were signs of dementia. To others it became evident during the assessment process as they performed poorly on the cognitive tests. A few participants did not associate their symptoms with impaired cognition and were unprepared for the outcome of the tests, as described in the following quotation.

*“My head was okay for a while but then it just became chaotic. And I believe that it has to do with my exhaustion and burn out symptoms, more than the other thing” (Participant 1, female).*

The participants that did describe that they were prepared for the outcome of the tests expressed a sense of relief as they gained an explanation for their symptoms. Others thought it would have been better not to know. Still, all participants, even those who felt prepared, experienced the diagnosis as a shock.

*“I was pretty convinced that I had it since I had the symptoms. I was completely convinced, and I was actually shocked that I reacted so strongly. That it affected me so deeply” (Participant 6, male).*

#### Personal and Social Life

Following the diagnosis, participants described the great impact it had on their personal lives. The diagnosis was experienced as a threat to their ability to fulfil certain obligations. They also imagined how their independence would be compromised as the disease progressed and how this could affect their lives and their possibilities to remain active and independent. One critical point anticipated by several participants was the day they would no longer be allowed to drive a car.

*“I did not think that I would react so strongly as I actually have done. I believe that they will take my driver´s license away from me. What are my chances then to go to the woods? If I don’t have a license and car, how can I get there? The forest is my life right now to just walk around” (Participant 4, female).*

The participants described how they were more sensitive to stress due to their cognitive symptoms and that daily activities were experiences as tiresome and stressful, and therefore needed extended recovery and rest affecting their social activities. Following the diagnosis, the participants and their family and friends, started to question their ability to maintain roles and responsibilities. This was related to how they perceived their own identity and the change from who they used to be to who they would become in relation to being a spouse or parent. Some experienced that family and friends changed their expectations of them, as illustrated by the next quotation.

*“I have always helped out in the kitchen, we all have been part of planning and cooking meals, except for my Grandma who is 82. But since my diagnosis I have been place beside Grandma, to keep her company not helping out anymore” (Participant 14, female).*

Participants with previous knowledge of dementia showed a greater insight into how their life may be affected. At the same time, all participants were aware the symptoms would gradually get worse, making them increasingly more dependent on others. The most important source of support were family and friends, as they in general were perceived to be understanding. Even so, the participants feared that family and friends might leave them. Therefore, some changed their own behavior to avoid disagreements or conflicts.

*“ I hold back so he won’t be annoyed with me… one does not hold the same position as before. That’s how I think anyway “oh dear I need to be extra nice now, for him to still want me” (Participant 10, female).*

#### Working Life

Several participants had stopped working before receiving the diagnosis, as their cognitive decline made it difficult to continue working. Support from employers varied, some participants described how they were forced to resign, while other described that their employers adjusted tasks and duties to meet their needs. Most participants who had to resign from work expressed missing working life and being part of a social context. For some, this loss was evident as they stated that they would have preferred to continue to work, with the right conditions.

*“Well, for most it come natural, one day we will all go, but for most it is voluntary when to say ”yes” it is time to leave this working life behind, to do something fun such as poking around in the garden, I love that. But it is a huge difference to be able to choose yourself or not to” (Participant 14, female).*

Being forced to stop working was experienced differently. Some were offered early retirement and others sick leave. Those offered sick leave often described complications with the Swedish Social Insurance Agency. The participants described the administrative staff to be unskilled and lack of understanding for the participants situation, leaving the participants with feelings of frustration. One consequence of this was that they did not receive their sickness benefit accordingly which for some meant financial problems and meant that some had to live of their lifesavings, causing distress and leading to practical consequences.

*“Well, concerning loans and things like that if you can’t get it you can’t fix the car. There are a lot of practical things that one is unable to do but I have to be calm and don’t stress myself” (Participant 12, female).*

### An Ongoing Process

Time was perceived as an important factor for the participants to comprehend their new situation. The participants reflected upon the time it took to process the diagnosis and how time was limited causing worries for the future. It became important to allow themselves time to reflect and to deal with feelings regarding their changed life situation.

#### Acceptance Takes Time

The participants emphasized the importance of allowing the process to take time. They described how thoughts of dementia occupied their mind most of the time after being diagnosed and how seeking information and learning more about dementia was important to gain understanding of their condition. Being diagnosed at an early phase, with only mild symptoms, sometimes made it more difficult to accept the disease.

*“I don’t know if I have faced the reality yet. I believe I am still in denial; it is an unreal feeling it is as simple as that. And I don’t know what this will lead I really don’t know, to face reality. To understand and to accept the disease before feeling ill is hard” (Participant 13, male).*

The participants reflected upon time in terms of delays, such as difficulties receiving an accurate diagnosis as symptoms and worries were not taken seriously. The participants described this process to be time consuming and of being stuck in time and not able to move forward.

*“It would be great if they (staff at the social welfare office) could follow the process somehow, as it is now, I am stuck. I can get anywhere” (Participant 1, female).*

#### Thoughts on Existence

According to participants, receiving the diagnosis had altered their view on life and what it may entail, especially regarding what to expect from time they have left. Most participants expressed stress as the uncertainty of how fast the disease would progress was undefined, fearing they would only have a few years left before becoming severely affected by dementia. For this reason, waiting several months for the next visit to the memory clinic caused distress among some participants.

*“The long wait of 8 months before seeing him (physicians) again is more like a year to me. It may be 5–10 years without but that can be 20% of the time I have left to live” (Participant 6, male).*

The participants also worried about how they as persons would change as the disease progressed. Some described a fear of one day no longer be able to participate as an active partner in family life or in society. Some participants reflected upon how a person with severe dementia might experience the world and expressed a fear of becoming a passive spectator, as the disease progressed.

*“I see it as being buried alive, that how I see it because it is like turning off the surrounding world. One can see and hear but not participate” (Participant 14, female).*

The anticipated changes were described as painful and difficult to grasp. This included the realization that they one day would be forced to leave their family. Thoughts about future losses was experienced as causing distress. Especially the thought of moving into a nursing home was unbearable to think about and often suppressed, as described in the following quote.

*“Well, and then when I have to move, I don’t know, I don’t want to think about it. I feels like well I have to stop the thought somehow. To think about it make me want to just disappear” (Participant 4, female).*

### Remaining in Control

The participants were aware that the disease gradually would make them lose control over their life and some reflected on alternative ways to stay in control and to avoid the later phases of dementia. At the same time the participants described how they tried to implement different coping strategies as means to remain in control for as long as possible.

#### To Tell or Not to Tell

Before disclosing the diagnosis to others, the participants described the need to contemplate and decide when in time and to whom they should tell. Some participants contacted others solely with the purpose to tell them about the diagnosis, while some brought it up in a casual conversation. The importance of choosing the right occasion was highlighted and the following quotation illustrates the importance of coming to terms with the diagnosis before telling others, especially in regard to telling their children.

*“I discovered that I can’t handle the situation yet and thought to myself that I am not the best bearer of news, I need to hold a straight back, be warm and cuddly and well prepared in order to tell them” (Participant 14, female).*

The participants who were transparent about the diagnosis described dementia as nothing to be ashamed of and that they hoped others might be more accepting if they knew. Nevertheless, by telling participants described how they no longer were in control of how information about them was shared. For those, choosing not to disclose their diagnosis several reasons were given, such as fear of being treated differently, not to cause worries and fear of being dismissed. It was also described as a strategy to protect themselves from receiving unwanted information about dementia.

*“I don’t want to tell her. I don’t want to listen to all her internet googles. I want to decide myself what I want to hear” (Participant 2, female).*

The risk of genetic and hereditary factors was addressed as they feared that their children would develop dementia in the future. This was for some a reason not to tell their children about the diagnosis.

*“They might dwell on it and wonder if they would catch it. It is unnecessary to start worrying about that now. To think about if it and if it is something we have in our family” (Participant 7, female).*

#### Information Needs and Preferences

Most of the participants reacted with shock when receiving the diagnosis and describe that it was difficult to absorb the information given by the physician. Not being prepared for the outcome of the memory evaluation also made it difficult to prepare and ask questions. Because of this, many questions were left unanswered. However, there were a few participants who was informed about the probability of dementia beforehand, these participants described how this increase the possibility for them to prepare questions for the physician.

*“As she told me about the results from the lumbar punction, she said that: “I will send you for a PET-scan, because the results indicate that you might have Alzheimer disease and I really want to examine you correctly”. That was the first indication that I might have it and then I started to realize what it meant as well as reading a lot about Alzheimer’s” (Participant 3, female).*

After receiving the diagnosis, the participants expressed difficulties finding information targeting young persons with dementia. At the time of diagnosis participants usually received a brochure about dementia available from the memory clinic. Several participants described the information in the brochure mainly to be targeting family caregivers and because of this some of them threw it away or gave it to family or friends. Some participants described an urgent need of more information and were active searching online. When seeking information online focus was primarily on disease progression, treatment, and possibilities to postpone further cognitive decline. To determine legitimacy of the information found online was challenging and were sometimes overcome by using reliable sources or verifying information by using multiple/other sources. Knowing more about disease progression was described as means to prepare for the future.

*“There is no reason for me to take something away. I know it is different for different people so it means that it might not affect me in the same way as others. But I try to take in all (information) I can and then I have to see how I process it and then decide to take one thing at the time” (Participant 5, female).*

Participants who did not seek additional information described different reasons for their decision. Some were satisfied with the information provided by the physician and were convinced that it was impossible to affect the outcome of the disease. Other participants described a fear of what they might find online and were hesitant to know about the later phases of dementia.

*“It is just awful to read about Alzheimer’s disease it is not joy whatsoever. So why should I read about it. It is what it is and me reading about it makes no difference at all” (Participant 10, female).*

#### Everyday Coping Strategies

Due to difficulties in preparing for or planning the future, many participants described how they decided to seize the day by focusing on activities that increased their wellbeing. This was also described as a way to distract themselves from thinking about the disease. For some participants spending time outdoors and being active was considered a good strategy to handle stress. To seize the day was described as living in the present and not postponing important things, as exemplified below.

*“In a way I am glad to have this diagnosis as I know that I only have this time and I can’t postpone thing. It is now or never, to seize the day and really enjoy time, and all the time think that well, tomorrow I might not be able to do this again” (Participant 7, female).*

To handle symptoms in everyday life participants described that they used notes and ‘planning ahead’ as strategies. These strategies were described as tools to reduce stress and irritation, both from others and themselves. The participants described different ways of preparing for the future, for example by teaching their partner to take over tasks, for example their private finances as illustrated in the quote.

*“We might need to change things. My responsibility has for example been to care for our finances’ a 100%. And he has no experiences of that at all so now we have decided that I need to show him how it is done, he needs to be in control of everything in case anything should happen to me” (Participant 3, female).*

The participants emphasized the need for more guidance from the memory clinic regarding everyday strategies, tools, and possibilities to delay the progression of dementia, as well as a need to meet other young persons with dementia. Some participants described how they tried to affect the outcome of the disease by making changes in lifestyle and behavior and being more active in cognitively challenging activities. The reasons for making these changes were mainly related to taking control over the progression of the disease, hoping to postpone cognitive decline.

*“I have not been physical active for some time and need to start again, I follow the diet of LCHF at the moment and will increase my intake of nice carbohydrates” (Participant 6, male).*

The medical treatment often offered following the diagnosis was described by the participants to offer a sense of hope. However, for some, it was hard to tolerate the side effects which made them consider pausing the treatment. Knowing that there is no cure for Alzheimer’s disease most participants described how they had a lot of faith in the treatment, hoping it would slow down the disease progression, and that they were therefore willing to endure the side effects.

*“Even if it can’t be cured it may slow the progression a bit. I am grateful for every week I get” (Participant 3, female).*

## Discussion

This study aimed to explore the experiences of persons recently diagnosed with dementia before the age of 65. The findings show that receiving such a diagnosis was experienced as a life changing moment, followed by them seeking to come to terms with the diagnosis and reflecting on its meaning, in which various strategies were adopted to maintain control. This study highlights three critical points considering the diagnosis of young-onset dementia that warrant special attention. It provides insight into factors may be associated with a delay in the transition process after receiving a young-onset dementia diagnosis, as well as factors that may facilitate transitions.

The most prominent critical point was receiving the diagnosis. Previous research has shown that persons with young-onset dementia have difficulties identifying themselves with the stereotype of a person with dementia (Rabanal et al., 2018). Participants in our study were not only relatively young, but also in an early stage of dementia, with only mild symptoms. This made it even more difficult for them to reorientate and integrate dementia into their lives, since being young and feeling vital, but also having an illness such as Alzheimer’s disease can seem contradictory. In addition, being middle-aged is associated with certain norms, competences, and expectations of the roles and responsibilities that are usually fulfilled. Dementia becomes an inevitable threat to those and receiving the diagnosis marks the beginning of a number of transitions in which several roles gradually will be lost.

A second critical point was related to the decision of telling others about the diagnosis. Being diagnosed with dementia due to Alzheimer´s disease made the participants question themselves and their abilities, resulting in feelings of no longer being equal to partners and friends. At the same time, the participants described that others changed their expectations and started treating them differently. Participants were thus confronted with their own stigma related to dementia, and with stigma by others. Research has shown that the stigma related to dementia might hinder persons from being open about their diagnosis out of fear of being treated in a different way (Milby et al., 2015). Although they also expressed this fear, most of our participants decided to be open about their diagnosis, hoping that others would be accepting and show greater understanding of their changed behavior and other symptoms. To consciously make decisions about who to tell or not to tell was used as a way of remaining in control of the information and lessen the risk of being treated as if they were “demented”.

A third crucial point identified in our study was the premature ending or working life. Most participants in our study left work abruptly, without any real closure. Most participants described a lack of support and understanding from employers. The importance of this critical point has previously been highlighted (Chaplin & Davidson, 2016; Evans, 2019; Greenwood & Smith, 2016; Richardson et al., 2016) and there are some studies on developing psychosocial interventions for persons with young-onset dementia, including programs for supervised work (Kinney et al., 2011; Robertson et al., 2013). Leaving their working life behind implied a sense of lost identity and loss of income and of meaningful activities. Previous research supports the loss of meaningful activities as important to address, as it seems to have a higher impact on well-being than the financial consequences (Greenwood & Smith, 2016). Our findings suggest that support should focus on maintaining both financial stability as well as meaningful and social activities, since financial difficulties, such as not receiving sickness benefits, were experienced as consuming energy and drew focus away from the process of accepting the diagnosis.

In addition, this study identified other factors that may influence and delay the process of a healthy transition. Such hindering factors can be linked to personal circumstances and the social environment or community, such as the access to services or reliable information (Meleis et al., 2000). In coherence with our findings, previous research has shown that symptoms of dementia among younger persons often are thought to be caused by stress or burnout, causing a delay in accurate diagnoses (Cabote et al., 2015; Greenwood & Smith, 2016). Participants in the present study also emphasized feelings of being left on their own to understand the provided information as it mainly focuses on older persons with dementia and family caregivers needs.

Other factors may facilitate a healthy transition and ease the process of accepting the diagnosis, access to reliable and adequate information being one important aspect (Meleis, 2010; Robinson et al., 2011). The participants in our study showed engagement in the transition process by seeking to stay in control and learning more about Alzheimer’s disease and how to delay further decline. This is in line with previous research that emphasize the need of information about the disease and its progression, how to deal with practical issues and accessible support strategies (Millenaar, Bakker, et al., 2016; Robinson et al., 2011; Sansoni et al., 2016). Further, the participants in our study described that they would have appreciated to attend group sessions with an educational purpose at the memory clinic. Such groups sessions would also offer an opportunity to meet other persons with dementia in similar life situations, which previously have been identified to be an important source of support and possibly decrease feeling of being lonely (Greenwood & Smith, 2016).

The interviews in our study were conducted within 2 months after receiving the dementia diagnosis, and the participants were most likely still in the midst of transitioning. This initial period after receiving the diagnosis is crucial as it offers a window of opportunity for timely support and guidance on how to integrate the implications of the diagnosis into their new reality and by that enhancing the possibility of a healthy transition (Meleis, 2010). Not knowing how the future will unfold and how fast the disease will progress, causes distress, and makes it difficult to plan ahead. All participants reflected upon how their freedom and independence would be affected the day they no longer would be allowed to drive or move into a nursing home. Previous research has highlighted the important role of healthcare professionals in acknowledging these internal psychological processes and emotional reflections that follow after diagnosis (Spreadbury & Kipps, 2019). More frequent contact with the clinic and conversations about these issues with professionals may enhance the possibility of a healthy transition, especially since our participants reported feelings of abandonment by healthcare services. Evidence from other fields, such as oncology, suggest for example that reassurance about non-abandonment by clinicians can reduce patients' emotional arousal and increase information recall of information provided in medical consultations (Sep et al., 2014; Visser et al., 2017). The importance of empathic communication by healthcare professionals and trusting healthcare professional-patient relationships is however not restricted to cancer care (Bensing et al., 2013). By providing continuous support and tailored information at a preferred pace, the person with dementia could better navigate through the period of reorientation after diagnosis, thereby reducing the risk of delays, distress and inadequate coping behavior that might prolong the transitions.

### Strengths and Limitations

There is a lack of studies that include younger persons living with dementia, and a clear strength of this study is that the persons themselves are given an opportunity to describe their experiences. However, there are some limitations that might affect the transferability of our findings. Unintentionally, all participants included in the study were diagnosed with dementia due to Alzheimer’s disease. Alzheimer’s disease is the most common cause of dementia, and among those with Alzheimer’s disease approximately four to six percent received their diagnosis before the age of 65 (Zhu et al., 2015). However, their experiences may differ from the experiences of persons with other types of dementia, because of differences in symptomology and disease progression. Another limitation is that we only included persons who could speak Swedish and had MMSE score of 24 or higher, which might impact the transferability of the findings to those with other ethnic backgrounds or severe dementia. In addition, only four of 14 participants were male, so it would be possible that there are gender differences in how dementia is experienced that we did not capture. Further, Johannessen et al. (2019) stated that the transition experiences of persons living with a partner might differ from the experiences of single people, as they are likely to receive support from their significant other. Most participants in our study were in a relationship, so it might be possible that the findings are not fully transferable to persons living alone. In addition, all participants were parents, in future studies it would be valuable to describe the perspectives of persons without children too. The inclusion from specialized memory clinics may not be representative for the population of younger persons with dementia living in more rural parts of Sweden, as there is a discrepancy as not all have the same access to specialized memory clinics and may receive the diagnosis in a later phase of the disease.

Future studies should include persons from different minority groups and contexts, with different types of dementia. In addition, to broaden our knowledge of how to facilitate transitions in young-onset dementia it is also important to investigate transitions and critical points in young-onset dementia from the perspective of healthcare professionals.

## Conclusion

Our findings describe the critical period after being diagnosed with young-onset dementia and highlight the need of support, adapted to the specific needs of younger persons. Coming to terms with the diagnosis and integrating it into one’s life is a process that takes time. To facilitate this process, persons could benefit from adequate information provision and acknowledgement of psychological processes and emotions provided by health care professionals, as well as support on how to handle practical and financial issues. Our findings suggest that by increasing knowledge and understanding of transitions, tailored support and reliable information can be provided at the most optimal time for young persons with dementia. As the tendency to seek information online is increasing, it is important that health care professionals are ready to meet the needs that follow this. This support might be especially important in the period after receiving the diagnosis, when the person is in a phase of reorientation. As the use of digital technology is increasing, it is also important to consider digital tools and online platforms as promising opportunities for providing support and social connection. It is also important to increase the knowledge and awareness of dementia outside the healthcare setting and to reduce the stigma. For example, employers could facilitate education regarding dementia and thereby increasing awareness. In addition, they could adapt work tasks to a person’s individual needs, supporting employees with dementia to continue work.

## References

[bibr1-14713012231155516] Alzheimer´s disease International (2019). World Alzheimer report 2019. Attitudes to dementia. https://www.alzint.org/resource/world-alzheimer-report-2019/

[bibr2-14713012231155516] Beattie A. Daker-White G. Gilliard J. Means R. (2004). ‘How can they tell?’ A qualitative study of the views of younger people about their dementia and dementia care services. Health & Social Care in the Community, 12(4), 359–368. 10.1111/j.1365-2524.2004.00505.x 15272891

[bibr3-14713012231155516] Bensing J. Rimondini M. Visser A. (2013). What patients want. Patient Education and Counseling, 90(3), 287–290. 10.1016/j.pec.2013.01.005 23395286

[bibr4-14713012231155516] Cabote C. J. Bramble M. McCann D. (2015). Family caregivers’ experiences of caring for a relative with younger onset dementia: A qualitative systematic review. Journal of Family Nursing, 21(3), 443–468. 10.1177/1074840715573870 25724671

[bibr5-14713012231155516] Chaplin R. Davidson I. (2016). What are the experiences of people with dementia in employment? Dementia, 15(2), 147–161. 10.1177/1471301213519252 24419354

[bibr6-14713012231155516] Clemerson G. Walsh S. Isaac C. (2014). Towards living well with young onset dementia: An exploration of coping from the perspective of those diagnosed. Dementia, 13(4), 451–466. 10.1177/1471301212474149 24339066

[bibr7-14713012231155516] Elo S. Kyngäs H. (2008). The qualitative content analysis process. Journal of Advanced Nursing, 62(1), 107–115. 10.1111/j.1365-2648.2007.04569.x 18352969

[bibr8-14713012231155516] Evans D. (2019). An exploration of the impact of younger-onset dementia on employment. Dementia, 18(1), 262–281. 10.1177/1471301216668661 27609937

[bibr9-14713012231155516] Graneheim U. H. Lundman B. (2004). Qualitative content analysis in nursing research: Concepts, procedures and measures to achieve trustworthiness. Nurse Education Today, 24(2), 105–112. 10.1016/j.nedt.2003.10.001 14769454

[bibr10-14713012231155516] Graneheim U. H. Lindgren B. M. Lundman B. (2017). Methodological challenges in qualitative content analysis: A discussion paper. Nurse Education Today, 56, 29–34. 10.1016/j.nedt.2017.06.002 28651100

[bibr11-14713012231155516] Greenwood N. Smith R. (2016). The experiences of people with young-onset dementia: A meta-ethnographic review of the qualitative literature. Maturitas, 92, 102–109. 10.1016/j.maturitas.2016.07.019 27621246

[bibr12-14713012231155516] Harris P. B. (2004). The perspective of younger people with dementia: Still an overlooked population. Social Work in Mental Health, 2(4), 17–36. 10.1300/J200v02n04_02

[bibr13-14713012231155516] Hendriks S. Peetoom K. Bakker C. van der Flier W. M. Papma J. M. Koopmans R. Verhey F. R. J. de Vugt M. Köhler S. Young-Onset Dementia Epidemiology Study Group Withall A. Parlevliet J. L. Uysal-Bozkir Ö. Gibson R. C. Neita S. M. Nielsen T. R. Salem L. C. Nyberg J. Lopes M. A. Dominguez J. C. Ruano L. (2021). Global prevalence of young-onset dementia: A systematic review and meta-analysis. JAMA Neurology, 78(9), 1080–1090. 10.1001/jamaneurol.2021.2161 34279544 PMC8290331

[bibr14-14713012231155516] Johannessen A. Engedal K. Haugen P. K. Dourado M. C. Thorsen K. (2019). Coping with transitions in life: A four-year longitudinal narrative study of single younger people with dementia. Journal of Multidisciplinary Healthcare, 12, 479–492. 10.2147/jmdh.S208424 31303758 PMC6605042

[bibr15-14713012231155516] Kinney J. M. Kart C. S. Reddecliff L. (2011). ‘That’s me, the Goother’: Evaluation of a program for individuals with early-onset dementia. Dementia, 10(3), 361–377. 10.1177/1471301211407806

[bibr16-14713012231155516] Kvello-Alme M. Bråthen G. White L. R. Sando S. B. (2019). The prevalence and subtypes of young onset dementia in Central Norway: A population-based study. Journal of Alzheimer’s Disease: JAD, 69(2), 479–487. 10.3233/jad-181223 31006688 PMC6598022

[bibr17-14713012231155516] Meleis A. I. Sawyer L. M. Im E. O. Hilfinger Messias D. K. Schumacher K. (2000). Experiencing transitions: An emerging middle-range theory. ANS. Advances in Nursing Science, 23(1), 12–28. 10.1097/00012272-200009000-00006 10970036

[bibr18-14713012231155516] Meleis A. I. (2010). Transitions theory : Middle range and situation specific theories in nursing research and practice. Springer Pub.

[bibr19-14713012231155516] Milby E. Murphy G. Winthrop A. (2015). Diagnosis disclosure in dementia: Understanding the experiences of clinicians and patients who have recently given or received a diagnosis. Dementia, 16(5), 611–628. 10.1177/1471301215612676 26493235

[bibr20-14713012231155516] Millenaar J. K. Bakker C. Koopmans R. T. Verhey F. R. Kurz A. de Vugt M. E. (2016). The care needs and experiences with the use of services of people with young-onset dementia and their caregivers: A systematic review. International Journal of Geriatric Psychiatry, 31(12), 1261–1276. 10.1002/gps.4502 27271788

[bibr21-14713012231155516] Millenaar J. K. de Vugt M. E. Bakker C. van Vliet D. Pijnenburg Y. A. Koopmans R. T. Verhey F. R. (2016). The impact of young onset dementia on informal caregivers compared with late onset dementia: Results from the NeedYD study. The American Journal of Geriatric Psychiatry: Official Journal of the American Association for Geriatric Psychiatry, 24(6), 467–474. 10.1016/j.jagp.2015.07.005 26560507

[bibr22-14713012231155516] Rabanal L. I. Chatwin J. Walker A. O’Sullivan M. Williamson T. (2018). Understanding the needs and experiences of people with young onset dementia: A qualitative study. BMJ Open, 8(10), e021166. 10.1136/bmjopen-2017-021166 PMC6196838 30344167

[bibr23-14713012231155516] Region Stockholm (2019). Regionalt vårdprogram 2019. Kognitiv sjukdom. https://vardgivarguiden.se/globalassets/kunskapsstod/vardprogram/kognitiv-sjukdom.pdf

[bibr24-14713012231155516] Richardson A. Pedley G. Pelone F. Akhtar F. Chang J. Muleya W. Greenwood N. (2016). Psychosocial interventions for people with young onset dementia and their carers: A systematic review. International Psychogeriatrics, 28(9), 1441–1454. 10.1017/S1041610216000132 27072752

[bibr25-14713012231155516] Robertson J. Evans D. Horsnell T. (2013). Side by side: A workplace engagement program for people with younger onset dementia. Dementia, 12(5), 666–674. 10.1177/1471301212473881 24337338

[bibr26-14713012231155516] Robinson L. Gemski A. Abley C. Bond J. Keady J. Campbell S. Samsi K. Manthorpe J. (2011). The transition to dementia—individual and family experiences of receiving a diagnosis: A review. International Psychogeriatrics, 23(7), 1026–1043. 10.1017/s1041610210002437 21281553

[bibr27-14713012231155516] Rose K. M. Lopez R. P. (2012). Transitions in dementia care: Theoretical support for nursing roles. Online Journal of Issues in Nursing, 17(2), 4. 10.3912/OJIN.Vol17No02Man04 22686112

[bibr28-14713012231155516] Samsi K. Manthorpe J. (2014). Care pathways for dementia: Current perspectives. Clinical Interventions in Aging, 9(default), 2055–2063. 10.2147/cia.s70628 25506210 PMC4259257

[bibr29-14713012231155516] Sansoni J. Duncan C. Grootemaat P. Capell J. Samsa P. Westera A. (2016). Younger onset dementia: A review of the literature to inform service development. American Journal of Alzheimer’s Disease and Other Dementias, 31(8), 693–705. 10.1177/1533317515619481 PMC10852741 26888862

[bibr30-14713012231155516] Sawchuk D. (2015). Aging and older adults in three roman catholic magazines: Successful aging and the third and fourth ages reframed. Journal of Aging Studies, 35, 221–228. 10.1016/j.jaging.2015.08.012 26568231

[bibr31-14713012231155516] Sep M. S. C. van Osch M. van Vliet L. M. Smets E. M. A. Bensing J. M. (2014). The power of clinicians’ affective communication: How reassurance about non-abandonment can reduce patients’ physiological arousal and increase information recall in bad news consultations. An experimental study using analogue patients. Patient Education and Counseling, 95(1), 45–52. 10.1016/j.pec.2013.12.022 24485947

[bibr32-14713012231155516] Socialstyrelsen (2014). Demenssjukdomars samhällskostnader i Sverige 2012. http://www.socialstyrelsen.se/publikationer2014/2014-6-3

[bibr33-14713012231155516] Spreadbury J. H. Kipps C. M. (2019). Measuring younger onset dementia: What the qualitative literature reveals about the 'lived experience' for patients and caregivers. Dementia, 18(2), 579–598. 10.1177/1471301216684401 28114802

[bibr34-14713012231155516] Visser L. N. C. Tollenaar M. S. de Haes H. C. J. M. Smets E. M. A. (2017). The value of physicians’ affect-oriented communication for patients’ recall of information. Patient Education and Counseling, 100(11), 2116–2120. 10.1016/j.pec.2017.06.005 28641989

[bibr35-14713012231155516] World Medical Association (2013). World medical association declaration of Helsinki: Ethical principles for medical research involving human subjects. JAMA, 310(20), 2191–2194. 10.1001/jama.2013.281053 24141714

[bibr36-14713012231155516] Zhu X. C. Tan L. Wang H. F. Jiang T. Cao L. Wang C. Wang J. Tan C. C. Meng X. F. Yu J. T. (2015). Rate of early onset Alzheimer’s disease: A systematic review and meta-analysis. Annals of Translational Medicine, 3(3), 38. 10.3978/j.issn.2305-5839.2015.01.19 25815299 PMC4356853

